# The maternal U1 haplogroup in the Koraga tribe as a correlate of their North Dravidian linguistic affinity

**DOI:** 10.3389/fgene.2023.1303628

**Published:** 2024-02-07

**Authors:** Jaison Jeevan Sequeira, Kadengodlu Vinuthalakshmi, Ranajit Das, George van Driem, Mohammed S. Mustak

**Affiliations:** ^1^ Department of Applied Zoology, Mangalore University, Mangalore, Karnataka, India; ^2^ Yenepoya Research Centre, Yenepoya (Deemed to be University), Mangalore, Karnataka, India; ^3^ Institut für Sprachwissenschaft, Universität Bern, Bern, Switzerland

**Keywords:** mtDNA, North Dravidian, Koraga tribe, Indus civilisation, tribes of India, Caucasus, last glacial maximum

## Abstract

**Introduction:** The Koraga tribe are an isolated endogamous tribal group found in the southwest coastal region of India. The Koraga language shares inherited grammatical features with North Dravidian languages. To seek a possible genetic basis for this exceptionality and understand the maternal lineage pattern, we have aimed to reconstruct the inter-population and intra-population relationships of the Koraga tribal population by using mtDNA markers for the hypervariable regions along with a partial coding region sequence analysis.

**Methods and Results:** Amongst the 96 individuals studied, we observe 11 haplogroups, of which a few are shared and others are unique to the clans Soppu, Oṇṭi and Kuṇṭu. In addition to several deep rooted Indian-specific lineages of macrohaplogroups M and U, we observe a high frequency of the U1 lineage (∼38%), unique to the Koraga. A Bayesian analysis of the U1 clade shows that the Koraga tribe share their maternal lineage with ancestral populations of the Caucasus at the cusp of the Last Glacial Maximum.

**Discussion:** Our study suggests that the U1 lineage found in the Indian subcontinent represents a remnant of a post-glacial dispersal. The presence of West Asian U1 when viewed along with historical linguistics leads us to hypothesise that Koraga represents a mother tongue retained by a vanquished population group that fled southward at the demise of the Indus civilisation as opposed to a father tongue, associated with a particular paternal lineage.

## 1 Introduction

The Indian subcontinent acted as a major corridor for early human migrations. Indian population structure has been shaped by various waves of migration, some of which followed a southern coastal route (Thangaraj et al., 2005). The most remarkable feature of Indian populations is the clear stratification between castes and tribes (Cordaux et al., 2003; Cordaux et al., 2004). The immense cultural, linguistic and ethnic diversity amongst Indian populations offers tremendous scope for genetic diversity studies. The tribal populations of India considered to be ‘aboriginal’ represent 8.6% of the total population (Registrar General and Census Commissioner of India, 2011). There are more than 700 tribes in India whose languages are either affiliated with the Austroasiatic, Indo-European, Dravidian and Trans-Himalayan languages families or represent linguistic isolates. In the broadest of geographical terms, Indian tribes tend to be classified as Southern, Northern, Eastern and Western tribal populations. Each ethnic tribal population is unique with respect to language, lifestyle and social customs. Studies have demonstrated that the populace of the Indian subcontinent is comprised of numerous small endogamous populations as a consequence of strict endogamy and social customs, resulting in the great complexity observed in the genetics of Indian populations (Cordaux et al., 2003; 2004; Thangaraj et al., 2005; 2006; Thanseem et al., 2006; Basu et al., 2016; Mustak et al., 2019). Several studies have highlighted that due to the founder effect, recessive diseases manifest themselves in Indian populations (e.g., Reich et al., 2009). The southern tribes have an interesting maternal haplotype distribution pattern with some tribes showing very high frequency of Indian-specific M haplogroup and others showing higher frequency of West Eurasian haplogroups (Anthropological Survey of India, 2021a). The Koraga tribe belongs to the latter group.

**FIGURE 1:**
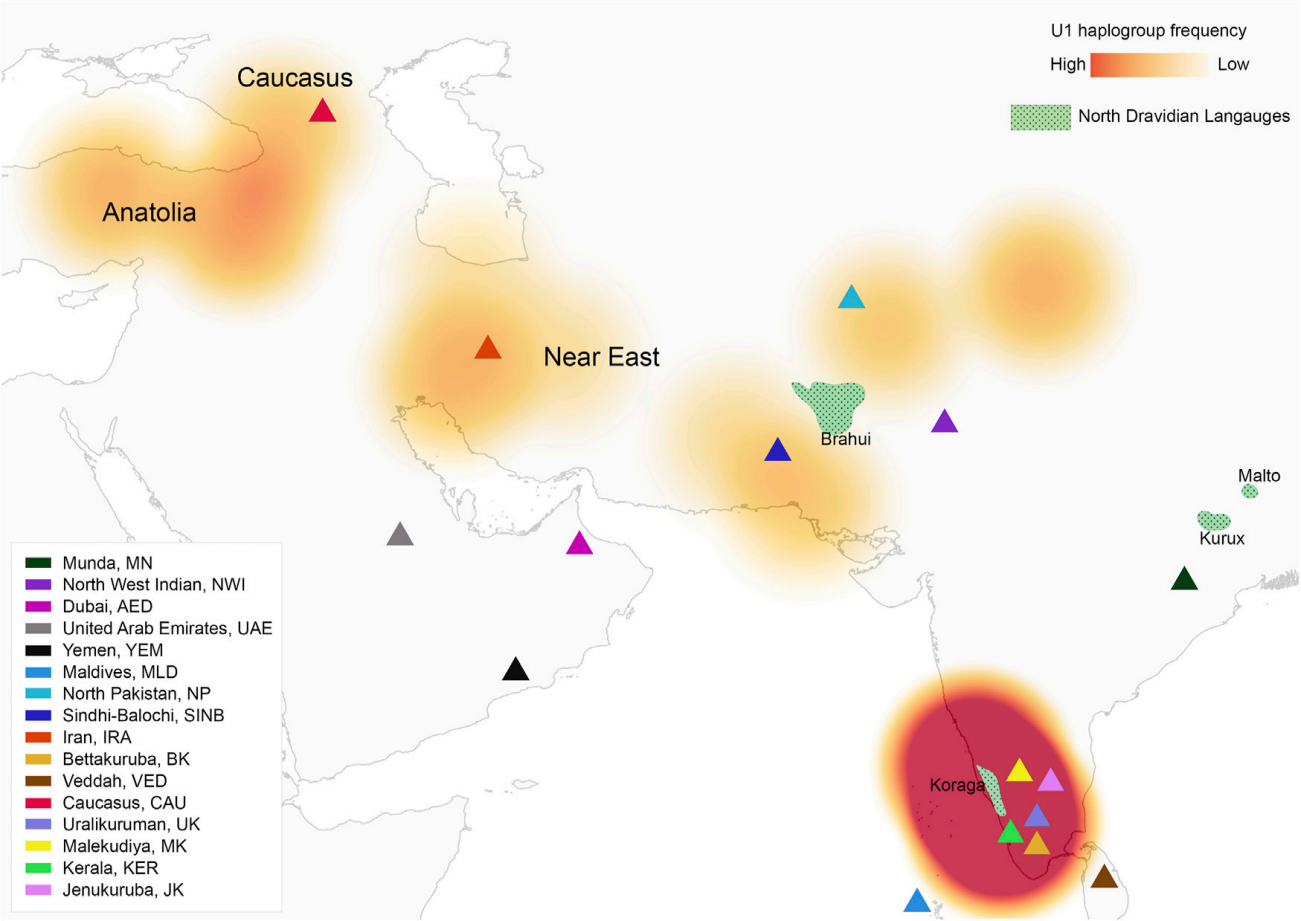
Map showing U1 haplogroup spatial distribution, geographical location of studied populations and North Dravidian languages.

The Koraga represent a small endogamous population with extremely low social status in Indian society. They are mainly concentrated in parts of Dakṣiṇa Kannaḍa and Uḍupi districts of Karnāṭaka, Kāsaragoḍ district of Kerala, and also found in small numbers in the adjoining districts of Śivamogga (Shimoga) and Koḍagu (Coorg) in Uttara Kannaḍa (Figure 1). As per the Census 2011, the Koraga population in these states tallies at 16,376. Between 1991 and 2011, a 10% decrease was observed in the number of individuals in Karnataka. The Koraga are amongst the poorest and most marginalised populations of South Kanarā. They weave baskets, cradles and winnowing trays, collect firewood and honey from nearby forests and work as seasonal labourers for a daily wage. Koraga people are classified into three endogamous groups, i.e., Soppu (ಸೊಪ್ಪು “lettuce”) or Tappu (ತಪ್ಪು “leaves”), Kuṇṭu (ಕುಂಟು “cloth”) and the Oṇṭi “earring” (Thurston and Rangachari, 1909). These divisions were based on the tribal dress they wore (Sherring, 1881). Bhat (1971) identified three dialect communities within the Koraga, which apparently coincide with the clan divisions, i.e., Tappu, ‘Mudu’ and Oṇṭi. The Koraga are traditionally classified within the Caṇḍāla caste. Progeny from the union of a higher caste female and a lower caste male are also classified within the untouchable Caṇḍāla category (Thurston and Rangachari, 1909). A folklore tale recounts a clash between Habāśika, a Koraga chieftain and his Caṇḍāla army from the Ghats, with the Kadamba rulers of Banavāsī (Walhouse, 1875).

The Koraga speak a Dravidian language, the precise phylogenetic propinquity of which within the language family remains unresolved. Bhat (1971) and McAlpin (1981) grouped Koraga together with Kurukh and Malto under the North Dravidian branch. Zvelebil (1990) proposed to treat Koraga as an independent branch of Dravidian under its own node in the tree, like Brahui. The Koraga language has been influenced for centuries by surrounding Tuḷu speakers, and many Koraga are bilingual in Tuḷu. Krishnamurti (2003) therefore grouped Koraga as close to Tuḷu and opined that Koraga look “like an offshoot of Tuḷu at a recent past.” However, he conceded that such a phylogenetic assignment would be problematic: “The location of Tuḷu in the family tree is doubtful and Koraga needs to be appropriately located in the subgrouping scheme.” At variance with Krishnamurti’s conjecture and in accordance with the phylogenetic assignments proposed by Bhat (1971), McAlpin (1981) and Zvelebil (1990), Koraga forms the past tense with the suffix <-k>, a grammatical feature which Koraga shares uniquely with North Dravidian and which is not found in South Dravidian languages.

The prehistory of Dravidian can only be unravelled against the backdrop of the caste system which arose in the aftermath of the advent of speakers of Indo-Aryan languages to the subcontinent at the beginning of the second millennium BC. The original *Śūdras* comprising the fourth and lowest caste or *varṇa* “colour” represented the vanquished indigenous pre-Aryan people who populated the Indus Valley civilisation before the Aryan invasion. However, the development of the caste system was a complex process, as were the patterns of migration set into motion by demographic changes connected to the decline of Harappan civilisation (van Driem, 2021). In this regard, Caldwell (1856) noted the significance of the fact that the majority of people in the Dravidian south were categorised as *Śūdras*. The vertical stratification of society determined the direction of migrations and linguistic assimilation but also the direction of hypergamy practised by women in communities of low status. Historically, the Koraga were considered to be amongst the lowliest of the untouchables. After Indian independence, positive measures were taken to provide social justice to historically underprivileged groups. Yet the legacy of low status outside of the caste system lingers on as a social stigma which endures to the present day.

Somatological impressionism in view of the physical phenotype prompted Stuart, in his report for the 1891 census, to opine that the “Koragas are an indigenous race. They speak Tuḷu language for communication with other people, but it is supposed they use a separate language at home quite distinct with any other main language family” (Stuart, 1893). Today the Koraga feel ashamed of their native language and immediately switch to Tuḷu if they notice that they are being observed by outsiders, even at a distance. The Koraga language has come to be perceived by the speakers themselves as an overt mark of their inferior socio-economic status in Indian society.

Various assumptions have been made about their origin by anthropologists, linguistics and historians, and origin stories exist in local folklore. Earlier genetic studies grouped the Koraga tribe under the South Indian tribes (Forster et al., 2002; Cordaux et al., 2003; Debnath et al., 2011; Gupta et al., 2012; Palanichamy et al., 2015; Anthropological Survey of India, 2021a). A recent genomic survey of over 2,000 samples from 75 Indian communities, mostly tribes, included Koraga genomes (Anthropological Survey of India, 2021b). The findings of these studies, although noteworthy, were confined to the broader objective of overall genetic diversity. A focused study interlinking linguistics and the maternal or paternal lineages of Koraga does not exist to date. The present study therefore aims to reconstruct the intra- and inter-population relationships of the Koraga tribe and provide an age estimate for the arrival of their ancestors on the southwestern coast of India, thereby correlating the findings with historical linguistics.

## 2 Materials and methods

The present study was approved by the Institutional Human Ethical Committee Mangalore University, Mangaluru (MU/AZ/349/IHEC/2014-2015 dated 15/07/2014). The purpose of the study was explained to all volunteers in vernacular language, and a written consent was obtained before sample collection. During the in-person interaction, their family history and medical history was obtained. Only those healthy individuals who were above the age of 18 and unrelated for at least three generations were included for blood sample collection. About 5–9 mL of intravenous blood sample was collected from 100 individuals from the Koraga tribal population belonging to different clans (Soppu, Kuṇṭu and Oṇṭi) and residing on the southwest coast of Karnataka and Kerala. This study was conducted in accordance with the Declaration of Helsinki**.** DNA was extracted using phenol-chloroform method described in Thangaraj et al. (2002). The extracted DNA was amplified using Applied Biosystems™ Veriti™ 96-Well Thermal Cycler with the following PCR conditions—95°C for 5 min followed by 35 cycles of 95°C for 30 s (denaturation), 52°C for 30 s (annealing) and 72°C for 60 s, and 72°C for 7 min (elongation). PCR products were sequenced with mitochondrial markers covering the control region (HVR-I and HVR-II) and partial coding region (see Supplementary Table S1). Sequences were compared with the revised Cambridge Reference Sequence (r-CRS), and haplogroups were assigned using Haplogrep 3 (Schönherr et al., 2023).

The mtDNA haplogroup analysis is the initial measure for identification of maternal lineage. Haplogroup frequency helps us to understand intra- and inter-population differences. In order to elucidate genetic differentiation within the Koraga population groups, Fst was estimated based on mtDNA haplogroup frequencies using Arlequin software (Excoffier and Lischer, 2010). Furthermore, PCA (principal component analysis) was performed using *prcomp* package in R software to understand the clustering pattern amongst selected populations based on the haplogroup frequencies. Median-joining network tree analysis was performed using the POPART software to assess haplotype sharing between the clans (Leigh and Bryant, 2015). Both PCA and median joining network analysis were performed with default parameters.

Bayesian analysis was performed using BEAST v2.7.4 software (Bouckaert et al., 2019) to measure the divergence time for U1 clades found in the Koraga and other global populations. We also included high-coverage ancient samples from the AmtDB database (Ehler et al., 2019) as calibration priors. Bayesian phylogenetic analysis was performed as per the protocol described by Connell et al. (2022). As an outgroup, the L2c2 mitogenome of a Moreno person (PaMOR16007) was employed. The sequences from the non-coding region were used in BEAST runs. Runs were performed using the HKY substitution model, and trees were connected. For the HVS1 and HVS2 regions, rigorous molecular clocks with mutation rates of 1.292 and 0.369 mutations/site/million years, respectively, were used (Connell et al., 2022). The following settings were used in Tree annotator to create a consensus tree: Common Ancestor Heights, 50% burning (produced higher posterior probability values). The tree was rerooted to the L2c2 outgroup in Figtree. In order to determine TMRCAs, median heights with 95% HPD (high probability densities) were used. All runs were carried out using 5,000,000 burning and 50,000,000 iterations, taking samples at intervals of 10,000 MCMC (Markov chain Monte Carlo) steps (Olivieri et al., 2017; Brandini et al., 2018; Capodiferro et al., 2021).

## 3 Results

### 3.1 Maternal haplogroup diversity within the Koraga tribe

**FIGURE 2:**
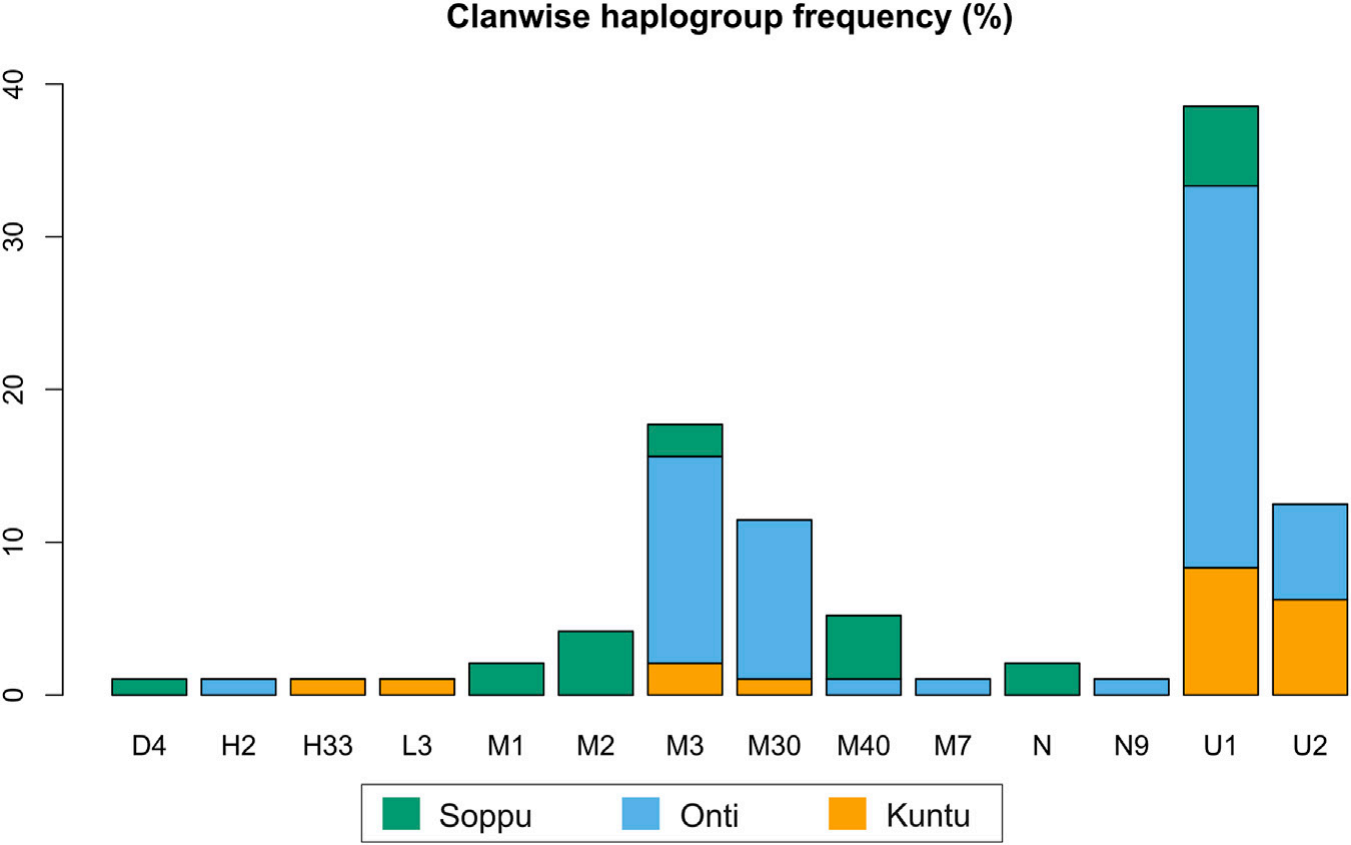
Clanwise haplogroup diversity in the Koraga tribe.

In the present study, mitochondrial markers were used to trace the maternal ethnic origin and population structure of the Koraga tribe. An earlier study by Cordaux et al. (2003) recorded only three major haplogroups, viz. U1a, M3 and U2a (lower frequency), with little diversity within the Koraga tribal population, whereas the present study identified the presence of 19 haplogroups, viz. L3e’i’k’x, U1, U1a, U1a1a, U2, U2a1, H2a*, H33b, M1a3, M1a3b1, M2a1a2, M3, M3a2a, M7a, M30, M40, N, N9b, D4k. However, the haplogroups U1a, U1a1a, U2a1, M3a2a and M30 accounted for 72% of the total variation. The previous study did not investigate clan-wise categorisation amongst the Koraga (Cordaux et al., 2003), whereas the present study categorised the Koraga into the three major clans Soppu, Kuṇṭu and Oṇṭi (Figure 2).

The haplogroups observed among the Koraga were nested in the lineages of macrohaplogroups M, N, R and U. Amongst the quantified mtDNA haplogroups, U1a1a (27.1%), M3a2a (16.7%), M30 (11.5%), U2a1 (9.4%) and U1a (7.3%) were the most common among the Koraga (Table 1). The majority of Koraga samples fell into known sub-lineages of mtDNA haplogroup U, viz. U1, U1a, U1a1a, U2 and U2a1, which altogether account for about 51% of the variation observed in West-Eurasian-specific lineages. Interestingly, U1 subclades uncommon to the Indian subcontinent (Kivisild et al., 1999) contributed to two thirds of the total U haplogroup distribution observed in the Koraga (Figure 1). The haplogroup U1a is characterised by an HVS1 back mutation at np16189 (T16189C!), most of the subclusters of which are defined by A385G (U1a1a) and C16400T in the non-coding region. C16400T has been reported earlier for the L, A, H and K subclades (van Oven and Kayser, 2009). We report C16400T for the U1a haplogroup in the Koraga population along with T199C. Autochthonous subclades of haplogroup U have been reported earlier for South Indian tribes (Ingman and Gyllensten, 2003; Sylvester et al., 2019) including the Koraga, i.e., U1a1a4, dated at 17,900 ± 6800 YBP (Palanichamy et al., 2015). In our study, the Koraga tribe show an unusually high frequency for U1 with a very low nucleotide diversity (*π* = 0.00064) and a relatively high haplotype diversity (Hd = 0.957), suggesting an early bottleneck, possibly towards the end of Late Glacial Maximum (∼18kya) (Silva et al., 2017) with the subsequent accumulation of variations (Grant and Bowen, 1998).

**TABLE 1:** Haplogroup distribution in the Koraga subgroups.

Origin	Haplogroup	Kuṇṭu	Oṇṭi	Soppu	Total (%)
West Eurasian	U1	0.0	7.0	0.0	4.2
	U1a	10.5	5.3	10.0	7.3
	U1a1a	31.6	29.8	15.0	27.1
African specific	L3e’i’k’x	5.3	0.0	0.0	1.0
	M1a3	0.0	0.0	5.0	1.0
	M1a3b1	0.0	0.0	5.0	1.0
	N	0.0	0.0	10.0	2.1
	N9b	0.0	1.8	0.0	1.0
Indian specific	M2a1a2	0.0	0.0	20.0	4.2
	M3	0.0	1.8	0.0	1.0
	M3a2a	10.5	21.1	10.0	16.7
	M7a	0.0	1.8	0.0	1.0
	M30	5.3	17.5	0.0	11.5
	M40	0.0	1.8	20.0	5.2
	U2	10.5	1.8	0.0	3.1
	U2a1	21.1	8.8	0.0	9.4
Others	D4k	0.0	0.0	5.0	1.0
	H2a*	0.0	1.8	0.0	1.0
	H33b	5.3	0.0	0.0	1.0
Total number of samples (*n*)		19	57	20	96

Haplogroups are classified based on their global distribution pattern.

Other than the West-Eurasian-specific mtDNA lineages, Indian-specific mtDNA lineages of haplogroup M and its subclades were also observed at a frequency of 41.7% amongst the Koraga (Table 1). We report the mtDNA haplogroups M2a1a2, M3, M3a2a, M7a, M30 and M40 in the Koraga in the current study. The approximate age estimates for these M haplogroups range from between 15 and 44 YBP (Chandrasekar et al., 2009), suggesting that the ancestors of the modern Koraga population admixed with individuals carrying Indian-specific haplogroups along their ancestral route of migration. M3 subclusters with a 17.7% frequency in the Koraga could indicate that admixture took place in the western or northwestern portion of the Indian subcontinent, since this haplogroup is predominant in these areas (Metspalu, 2004). Another interesting finding is the presence of traces of the African L3e’i’k’x, M1a3 and M1a3b1 haplogroups in the Koraga population. Although retained only as traces, these haplogroups represent unambiguous artefacts of the earliest human migrations out of Africa (Olivieri et al., 2006).

Some mitochondrial haplogroups were shared amongst the three clans, whilst some were unique to a particular clan. The mtDNA haplogroup U1a and its subclades are shared by all three Koraga clans and distributed almost equally amongst them (5.3%–31.6%). Haplogroup M3a2a was the second most common haplogroup, likewise shared amongst all three Koraga clans (Table 1). The haplogroup U2a1 was found in the highest frequency in the Kuṇṭu at 21.1%, at a lower frequency in the Oṇṭi at 8.8%, and completely absent in the Soppu Koraga. The haplogroup M2a1a2 was found only in the Soppu, at a frequency of 20%, and absent in the Kuṇṭu and Oṇṭi. The haplogroup M30 was absent in the Soppu, whereas M40 was found to be more frequent in the Soppu as compared to the Kuṇṭu and Oṇṭi. Overall, the Kuṇṭu reported a higher frequency of West Eurasian U1 subclusters (42.1%), whilst the Soppu reported a higher frequency of Indian-specific M (60%) (Table 1).

**FIGURE 3:**
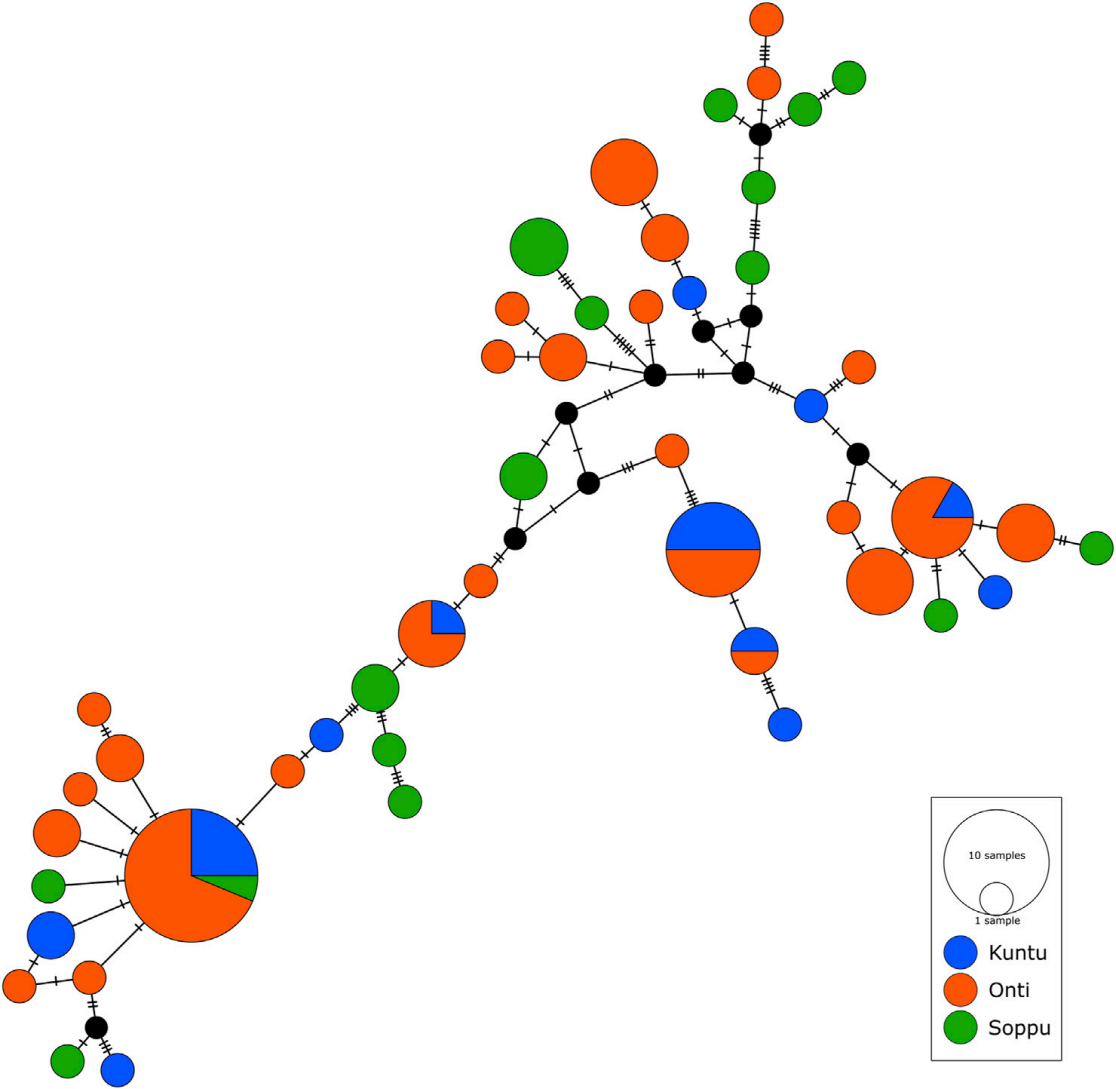
Median joining network showing haplotype sharing pattern between the clans. Each node represents a unique mtDNA haplotype. Size of the node represents relative haplotype frequency. Each hatch mark/bar represents a nucleotide change. Black nodes represent unsampled or extinct ancestral sequence.

We performed network analysis to understand the haplotype sharing pattern between the clans. In the 96 samples studied, 48 haplotypes were reported. The Oṇṭi and Soppu clans exhibit more unique haplotypes, whereas most of the haplotypes in the Kuṇṭu are shared (Figure 3). The lowest haplotype diversity was observed in the Oṇṭi and the highest in the Soppu. The African-specific haplogroups M1 and N occur uniquely in the Soppu clan. The Indian-specific haplogroups M2 and M40 are found at higher frequencies in the Soppu than in the other two clans, whilst the haplogroups U1 and M3 are evenly present in all the three clans, all of this suggesting a population stratification within the tribe.

**FIGURE 4:**
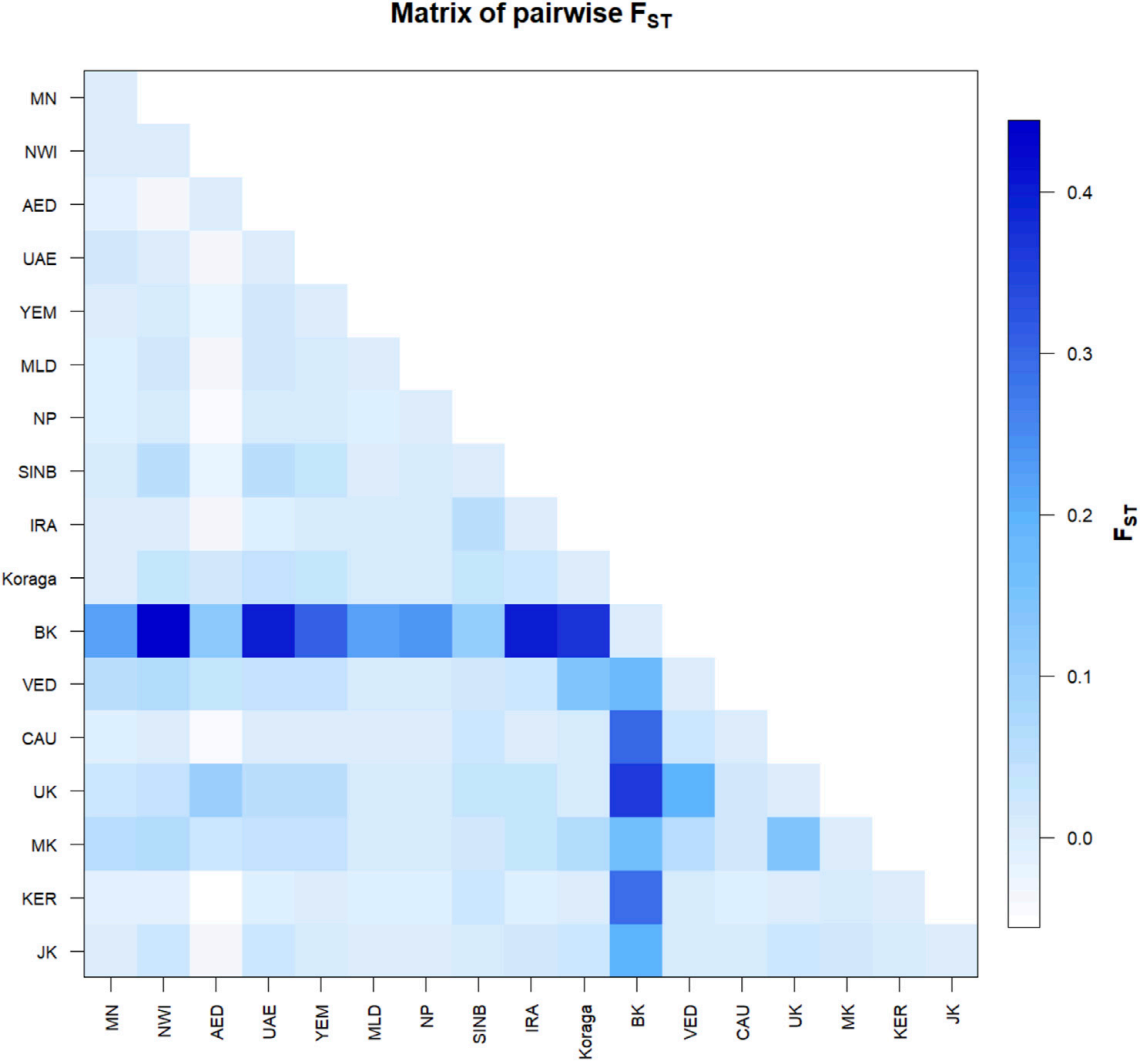
Pairwise Fst variation in the Koraga tribe and other populations spread along areas of U1 haplogroup prevalence. MN, Munda; NWI, North West Indian; AED, Dubai; UAE, United Arab Emirates; YEM, Yemen; MLD, Maldives; NP, North Pakistan; SINB, Sindhī-Balocī; IRA, Iran; BK, Bëṭṭʉ Kuṟumba; VED, Vedda; CAU, Caucasus; UK, Ūrāḷi Kuruman; MK, Malekuḍiya; KER, Kerala; JK, Jēnu Kuṟumba.

The pairwise F_st_ values for the clans lie between 0.02 and 0.12, suggesting that the Koraga clans are less differentiated within the population (Supplementary Figure S1). The Soppu clan is less closely related to the Kuṇṭu than to the Oṇṭi (Supplementary Figure S1). When the Koraga population is compared with other populations, F_st_ was in the range of 0.10–0.45 (Figure 4), suggesting that the Koraga population is more isolated. Such isolation may have preserved the original genetic variation more faithfully, as is evident from the diversity indices (Table 2). Because of a strong founder effect, a reduced nucleotide diversity is observed within this population (Tournebize et al., 2022). This founder effect is also reflected in their higher genetic distance as well as their higher average number of pairwise differences (Supplementary Figure S2). The F_st_ analysis indicates that the Koraga tribe are a drifted population due to higher pairwise differences with the neighbouring tribes (Cordaux et al., 2003).

**TABLE 2:** Diversity indices for Koraga clans.

Clan	Haplotype diversity (Hd)	Nucleotide diversity (π)
Kuṇṭu	0.9240	0.000553
Oṇṭi	0.9417	0.000607
Soppu	0.9737	0.000700
Overall	0.957	0.00064

### 3.2 Koraga in comparison with West Asian, Caucasian and South Indian tribes

In the inter-population pairwise F_st_ analysis, we observe that the average pairwise distances between Koraga and other populations (Supplementary Figure S2 above the diagonal) are comparable with those of the Ūrāḷi Kuruman (UK) and Malekuḍiya (MK) tribes. The population pairwise distances are the lowest in the Koraga, followed by the Ūrāḷi Kuruman. Two populations, namely, the Bëṭṭʉ Kuṟumba (BK, cf. Zvelebil, 1982) and the Sindhī-Balocī (SINB), stand out with their higher genetic distances. Interestingly, the Nei’s distance between the Koraga and the Sindhī-Balocī, a northwestern population, is less than between the Koraga and the South Indian Bëṭṭʉ Kuṟumba tribe. Similarly, the F_st_ value between the Koraga and Caucasian populations is 0.01 (Figure 4), which is much less than between the Koraga and the Bëṭṭʉ Kuṟumba (0.36), suggesting that the Koraga exhibit a greater maternal affinity with populations of the Caucasus and West Asia than with Indian tribes rich in the M2 haplogroup.

**FIGURE 5:**
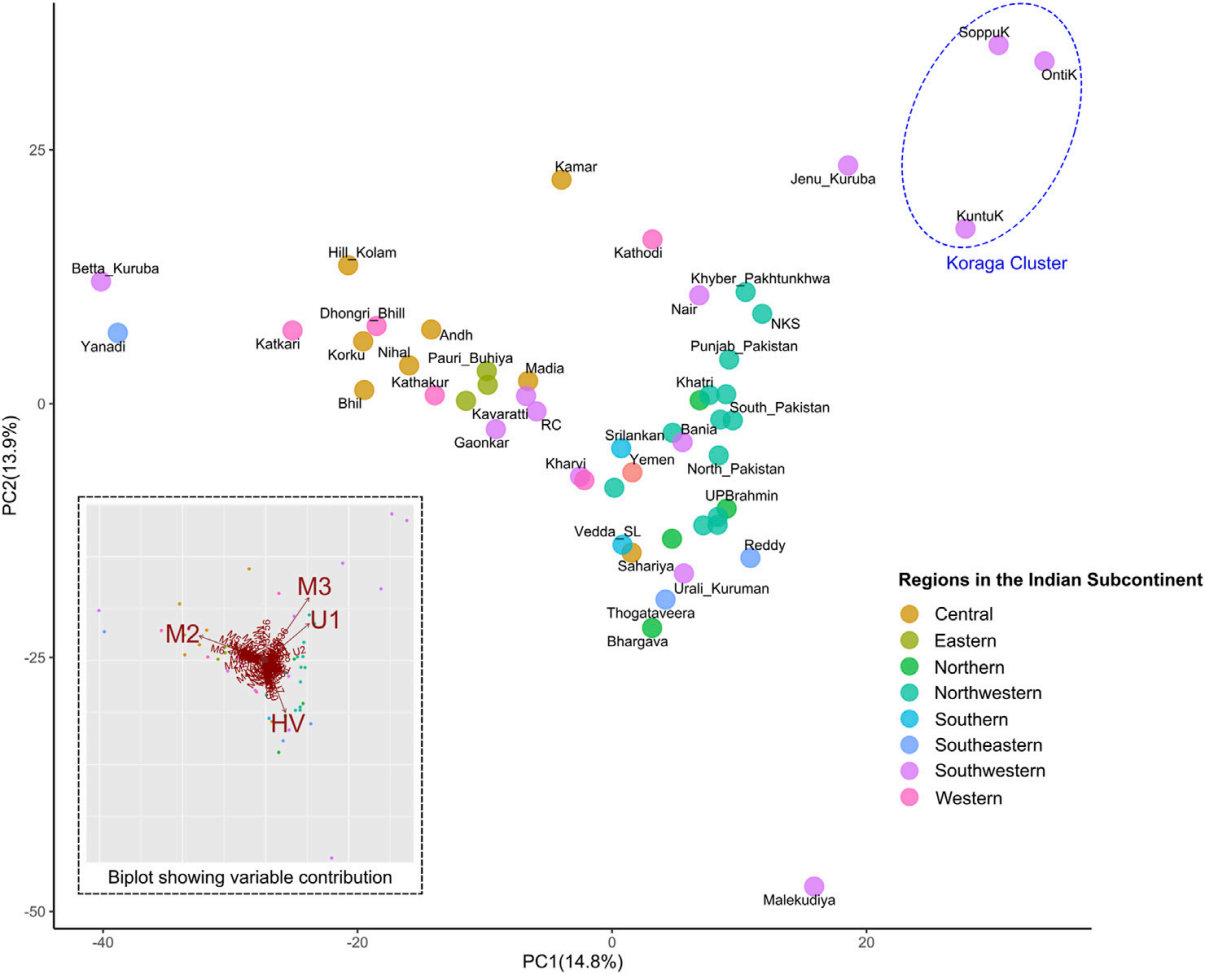
PCA showing clustering pattern in the Koraga tribe and other populations from the Indian subcontinent. Inset has a biplot showing haplogroups with highest contribution to the variance. Koraga cluster is encircled in a dotted ellipse.

Furthermore, in order to understand the clustering pattern with other populations, Principal Component Analysis (PCA) was performed using the haplogroup frequencies (Figure 5). Populations were chosen based on prior information concerning their regional affiliation. The Koraga formed a separate cluster, closer to the northwestern populations. The only other southern population closer were the Jēnu Kuṟumba. The biplot showed that the formation of this cluster was mainly due to the U1 and M3 haplogroups present in these tribes.

Earlier studies observed that the influence of West Eurasian mtDNA haplogroups is greater amongst high-caste populations such as the Brahmins, and amongst Muslims, whereas the frequencies of West Eurasian haplogroups were reportedly lower amongst tribal populations (Bamshad et al., 2001; Roychoudhury et al., 2001; Kivisild et al., 2003). In contrast to previous studies, the present study shows the prevalence of West Eurasian mitochondrial haplogroup to be high in the Koraga. Although haplogroup U1 is responsible for this contrast, the contribution of M3 in both the Koraga and Jēnu Kuṟumba (a.k.a. Kāṭṭu Nāyakkar, Kaṭṭunāyakan, cf. Zvelebil, 1988) should not be ignored, as this haplogroup is widely present in northwestern populations carrying the Ancestral North Indian component (Reich et al., 2009). With the absence of Y haplogroups associated with recent migration and admixture in both these tribes (Anthropological Survey of India, 2021c), it is unlikely that the gene pool of these tribes was in any way influenced by recent demographic changes. Therefore, the movement of M3 or U1 must have resulted from an earlier wave of migration. Previous studies have, in fact, suggested that the spread of Neolithic agriculture was associated with the pre-Bronze-Age migration of West Eurasian haplogroups into South India (Kivisild et al., 1999; Palanichamy et al., 2004). Others have associated the presence of HV and U1 haplogroups in South India with a proto-Dravidian migration (Palanichamy et al., 2015). We too, observed a close relationship with the Iranian U1 sequences and the Koraga U1 sequences in the Neighbour Joining tree (Supplementary Figure S3).

### 3.3 Divergence time estimate for U1 haplogroup in the Koraga tribe

**FIGURE 6:**
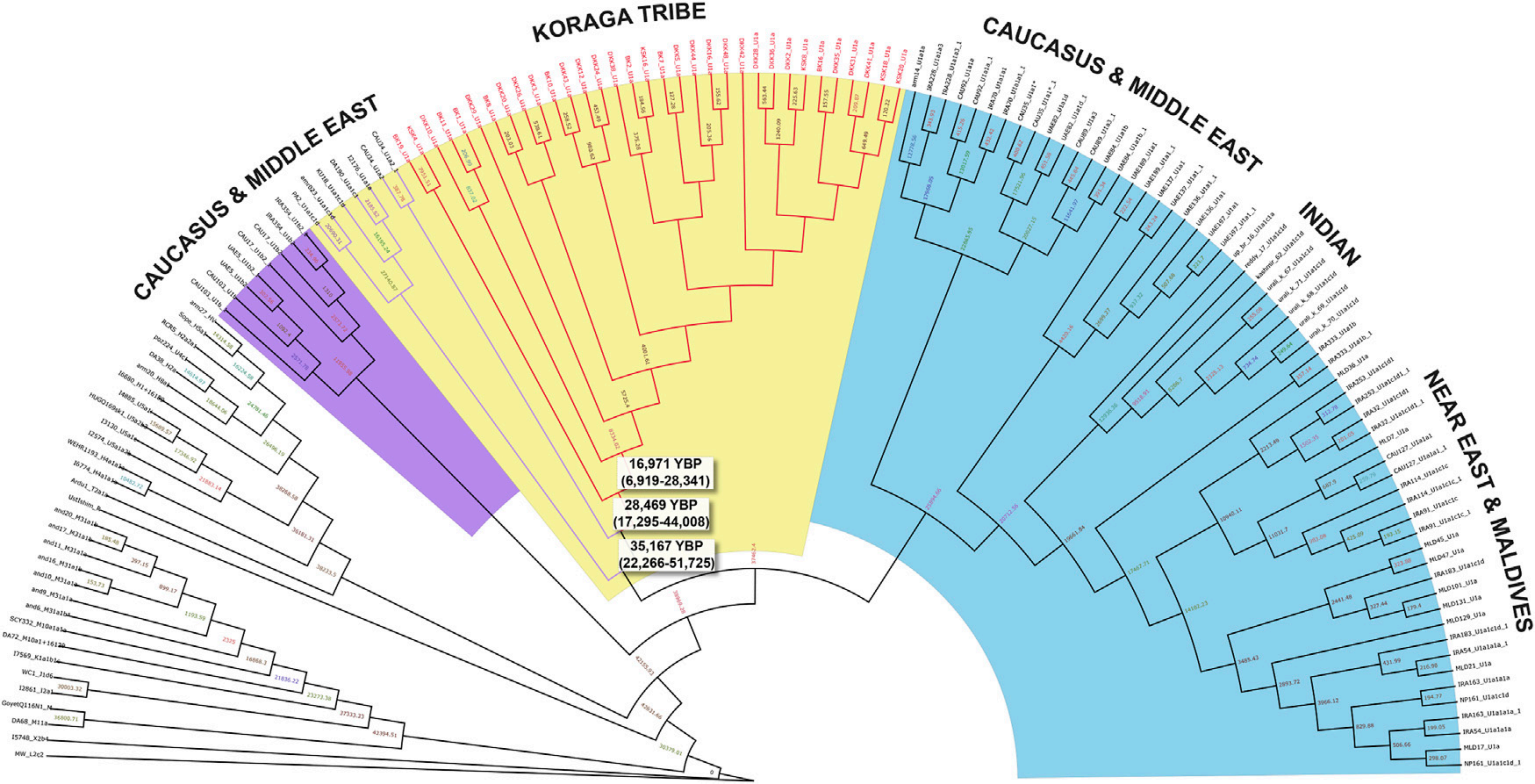
Bayesian phylogenetic tree for U1 haplogroup. Divergence time estimates are shown for the U1 samples found in the Koraga tribe (red branches) and Caucasus and Middle Eastern samples (purple branches).

In order to date the U1 cluster found in the Koraga tribe, we performed Bayesian analysis. In the Bayesian phylogenetic tree constructed for the maternal U1 haplogroup (Figure 6), all Koraga individuals clustered under the single clade U1a, a maternal lineage shared with populations of the Caucasus, dating from ∼28,000 years ago. The next divergence happened in the LGM recovery period, resulting in a TMRCA dating from ∼16,000 years ago. All the U1 subclades of the Koraga tribe can be presumed to have arisen from a founder whose descendants reached the southwestern coast of the Indian peninsula. These assumptions are based on the spatial distribution of U1 haplogroup (Figure 1) and the divergence time gradient observed in U1 clades from the Caucasus to South India. The TMRCA measured in our study is consistent with earlier results (Supplementary Table S2).

## 4 Discussion

As with the Hungarians, whose language provides unambiguous evidence of the provenance of the language community notwithstanding the complexity of the Hungarian gene pool (van Driem, 2021), so too the Koraga language clearly indicates a North Dravidian provenance of the community. The genetic profile of the Koraga reveals not just the complex history of the language community but also sheds a new light on the ethnolinguistic prehistory of the Indian subcontinent. Uniparental markers may reveal different pasts of a single language community. Poloni et al. (1997), Poloni and Nicolas Ray (2000) reported that globally the distribution of language families patterned geographically with the prevalent paternal lineages of language communities. Subsequent studies showed how ubiquitously ethnolinguistic phylogeography correlated with Y chromosomal haplogroups, but also stressed that these correlations were neither perfect nor universal (van Driem, 2013; van Driem, 2021). The Bodish language communities of Baltistan in Pakistan-occupied Kashmir (Zerjal et al., 1997; Quintana-Murci et al., 2001; Qamar, 2002; van Driem, 2014) and the Laccadives (Mustak et al., 2019) both represent salient cases of a mother tongue correlation at variance with the globally more frequent father tongue correlation. Palanichamy et al. (2015) suggested that maternal molecular markers might serve as tracer dyes for the spread of Dravidian, and we argue that their proposal makes sense for the Koraga in light of the known sociolinguistic history of the Indian subcontinent.

Paternal lineages of a community afflicted with low social status are less likely to fare well over time. The Brahui are a North Dravidian language community in Beluchistan, who are conventionally regarded as an *in situ* linguistic remnant of the Dravidian populace of the Indus civilisation. It was proposed that the paternal lineage L might serve as a molecular tracer for the ancient spread of Elamo-Dravidian (van Driem, 2012). However, the Beluch exhibit haplogroup L at a higher frequency than any other group in Pakistan, more so than the Brahui (Qamar, 2002; Kivisild et al., 2003; Trivedi, 2008; Haber et al., 2012; Lacau et al., 2012). This paradoxical finding represents an expected outcome of the social stigma connected with the pre-Indo-Aryan ethnolinguistic identity, which rendered the Brahui prone to genetic contribution from Aryan males through hypergamy practised by succeeding generations of Brahui women (van Driem, 2012; 2021). As in the case of Hungarian, the Brahui language community represents a purely linguistic retention (Pagani et al., 2017). On the other hand, the paternal lineage L (M76) preserved in the Liṅgāyat, Okkaliga and other agriculturalist population groups trace an ancient pathway of southward migration from the Indus basin along the southwestern coast.

As with Brahui, the language of the Koraga served as the most conspicuous marker of inherited low social status, and an earlier study on the Koraga argued that the low haplotype and nucleotide diversity in the tribe indicated strong genetic drift (Cordaux et al., 2003). The mitochondrial lineage U1 observed in the Koraga clusters phylogenetically with populations of Western Asia and the Caucasus (Palanichamy et al., 2015). Tribal groups in South India exhibit reduced diversity and large genetic distances, both among themselves and as compared with other groups. These results reveal no signals of prehistoric demographic expansion and instead reflect enhanced genetic drift, to which these groups were subjected due to small population size and/or bottlenecks.

The Y haplogroup analysis from an earlier study revealed that the Koraga differ markedly from other tribes and caste populations. Y-chromosomal haplogroup D, characterised by the Y-chromosome Alu insertion polymorphism (YAP) at locus DYS287, occurs in a trace amount in the Koraga tribe (Cordaux et al., 2004). This paternal lineage represents a remnant of a very early spread eastward across South Asia, with areas of retention today on the Andamans, in the Himalayas, parts of Southeast Asia and the Japanese archipelago, especially in the Ainu and the Ryūkyūans (Chandrasekar et al., 2007; van Driem, 2021). More salient is the exceedingly high frequency of Y-chromosomal haplogroup H1 (M82) in the Koraga (Anthropological Survey of India, 2021c), a paternal lineage also found in high frequency in Gōṇḍ tribes (Sharma, 2009), in the Kātkarī and Jēnu Kuṟumba and other population groups categorised as untouchable or of low status (Anthropological Survey of India, 2021c). The frequency of Y chromosomal haplogroup F is particularly high in the Sōliga and Mādiga, two untouchable ‘scheduled’ castes who have assimilated to the Dravidian language communities surrounding them and who now each speak their own dialect of Kannaḍa and Telugu respectively. The age of haplogroup F has been estimated at ∼25,000 YBP, reflecting an older stratum of population, whilst the expansion of haplogroup H in the Indian subcontinent has been estimated at ∼7,000 YBP (Poznik et al., 2016).

The only other paternal haplogroup found in the Koraga tribe is R2 (M124), which may reach a frequency of up to 40% in some other Dravidian tribal groups. The Siddi tribe, which is of historically comparatively recent African origin and shows about 40% YAP polymorphism (Thangaraj et al., 1999), is found in geographical proximity to the Koraga population. Some gene flow between the two seems possible, however, the African-specific haplogroups like M1 observed in the Koraga have not been reported in Siddi tribes before (Shah et al., 2011).

The mitochondrial clade U1 in the Koraga tribe ultimately originates in West Asia, and a close affinity between the Caucasian U1 and the Koraga U1 cluster is observed. Based on our Bayesian estimate, the movement of U1 maternal ancestors began at the time of the Last Glacial Maximum. Similar West Eurasian founder lineages are reported in the Ūrāḷi Kuruman, Malekuḍiya, Lakṣadvīp Islanders and other tribes on the southwestern coast (Forster et al., 2002; Palanichamy et al., 2015; Mustak et al., 2019; Sylvester et al., 2019). This maternal lineage may have been borne southward into the Indian subcontinent in the aftermath of the demise of the Harappan civilisation. The presence of West Eurasian mitochondrial haplogroups U1, HV and U7 elsewhere in the Dravidian heartland suggests different waves of incomers to South India at different time depths. The Koraga did not experience recent gene flow from neighbouring tribes, but the two sets of uniparental markers in the language community capture two divergent facets of the Koraga past.

In conclusion, the untouchable status of the Koraga language community, early somatological impressionism based on physical phenotype, the septentrional phylogenetic position of the language within the Dravidian language family in combination with the contrast between our mitochondrial findings and the paternal lineages borne by the population allow us to present the hypothesis that Koraga is a mother tongue retained by a vanquished population group that fled southward at the demise of the Indus civilisation. The original Koraga migrant group encountered other Dravidian populations whose linguistic ancestors had preceded them as part of a pre-Bronze Age southward dispersal of Elamo-Dravidian languages. The reviled social status of the Koraga language community doomed the long-term survival prospects of the original Koraga paternal lineages and enabled their replacement by paternal lineages introduced into the community from local untouchable populations, whereas only the Koraga maternal lineage retained an ancestral correlation with the linguistic affiliation of the language community. This situation has two parallels in the Brahui and Kurukh, where the native Y chromosomes were lost or reduced through hypergamy practised by Dravidian women and Munda women respectively, marrying local men of the Indo-Aryan (i.e., Beluch) and Austroasiatic language communities (Chaubey et al., 2011; van Driem, 2012). This pattern permits us to infer the original low status of Northern Dravidian speakers as a consequence of their subjugation during the demise of the Indus civilisation.

## Data Availability

The original contributions presented in the study are included in the article/Supplementary Material, further inquiries can be directed to the corresponding authors.
