# Corrigendum: The maternal U1 haplogroup in the Koraga tribe as a correlate of their North Dravidian linguistic affinity

**DOI:** 10.3389/fgene.2024.1384937

**Published:** 2024-03-12

**Authors:** Jaison Jeevan Sequeira, Kadengodlu Vinuthalakshmi, Ranajit Das, George van Driem, Mohammed S. Mustak

**Affiliations:** ^1^ Department of Applied Zoology, Mangalore University, Mangalore, Karnataka, India; ^2^ Yenepoya Research Centre, Yenepoya (Deemed to be University), Mangalore, Karnataka, India; ^3^ Institut für Sprachwissenschaft, Universität Bern, Bern, Switzerland

**Keywords:** mtDNA, North Dravidian, Koraga tribe, Indus civilisation, tribes of India, Caucasus, last glacial maximum

In the published article, there was an error in the legend for **Figure 4** as published. The following part was incorrect: “SINB, Sindhi-Baloci; IRA, Iran; BK, Betta Kuruba; VED, Vedda; CAU, Caucasus; UK, Urali Kuruman; MK, Malekudiya; KER, Kerala; JK, Jenu Kuruba.” The corrected legend appears below.

“FIGURE 4 | Pairwise Fst variation in the Koraga tribe and other populations spread along areas of U1 haplogroup prevalence. MN, Munda; NWI, North West Indian; AED, Dubai; UAE, United Arab Emirates; YEM, Yemen; MLD, Maldives; NP, North Pakistan; SINB, Sindhī-Balocī; IRA, Iran; BK, Bëṭṭʉ Kuṟumba; VED, Vedda; CAU, Caucasus; UK, Ūrāḷi Kuruman; MK, Malekuḍiya; KER, Kerala; JK, Jēnu Kurumba.”

In the published article, there was an error. “Sindhi-Balochi” should be “Sindhi-Baloci” and “Betta Kurumba” should be: Bëṭṭʉ Kuṟumba.”

A correction has been made to **Results**, *3.2 Koraga in comparison with West Asian, Caucasian and South Indian tribes*, Paragraph number 1. This sentence previously stated:

“Two populations, namely, the Betta Kurumba (BK, cf. **Zvelebil, 1982**) and the Sindhi-Baloci (SINB), stand out with their higher genetic distances. Interestingly, the Nei’s distance between the Koraga and the Sindhi-Baloci, a northwestern population, is less than between the Koraga and the South Indian Betta Kurumba tribe. Similarly, the F_st_ value between the Koraga and Caucasian populations is 0.01 (**Figure 4**), which is much less than between the Koraga and the Beṭṭa Kuṟumba (0.36), suggesting that the Koraga exhibit a greater maternal affinity with populations of the Caucasus and West Asia than with Indian tribes rich in the M2 haplogroup.”

The corrected sentence appears below:

“Two populations, namely, the Bëṭṭʉ Kuṟumba (BK, cf. **Zvelebil, 1982**) and the Sindhī-Balocī (SINB), stand out with their higher genetic distances. Interestingly, the Nei’s distance between the Koraga and the Sindhī-Balocī, a northwestern population, is less than between the Koraga and the South Indian Bëṭṭʉ Kuṟumba tribe. Similarly, the F_st_ value between the Koraga and Caucasian populations is 0.01 (**Figure 4**), which is much less than between the Koraga and the Bëṭṭʉ Kuṟumba (0.36), suggesting that the Koraga exhibit a greater maternal affinity with populations of the Caucasus and West Asia than with Indian tribes rich in the M2 haplogroup.”

A correction has been made to **References**. This sentence previously stated:

“Zvelebil, K. V. (1982). Bëttukurumba: first report on a tribal language. J. Am. Orient Soc. 102, 523. doi:10.2307/602307.”

The corrected sentence appears below:

“Zvelebil, K. V. (1982). Bëṭṭʉ Kuṟumba: first report on a tribal language. J. Am. Orient Soc. 102, 523. doi:10.2307/602307.”

In the published article, there was an error. “South-India” should be “South India.”

A correction has been made to **Results**, *3.3 Divergence time estimate for U1 haplogroup in the Koraga tribe*, Paragraph number 1. This sentence previously stated:

“These assumptions are based on the spatial distribution of U1 haplogroup (**Figure 1**) and the divergence time gradient observed in U1 clades from the Caucasus to South-India. The TMRCA measured in our study is consistent with earlier results (**Supplementary Table S2**).”

The corrected sentence appears below:

“These assumptions are based on the spatial distribution of U1 haplogroup (**Figure 1**) and the divergence time gradient observed in U1 clades from the Caucasus to South India. The TMRCA measured in our study is consistent with earlier results (**Supplementary Table S2**).”

In the published article, there was an error. “Jēnu Kurumba” should be “Jēnu Kurumba.”

A correction has been made to **Results**, *3.2 Koraga in comparison with West Asian, Caucasian and South Indian tribes*, Paragraph number 2. This sentence previously stated:

“The Koraga formed a separate cluster, closer to the northwestern populations. The only other southern population closer were the Jēnu Kurumba.”

The corrected sentence appears below:

“The Koraga formed a separate cluster, closer to the northwestern populations. The only other southern population closer were the Jēnu Kuṟumba.”

A correction has been made to **Results**, *3.2 Koraga in comparison with West Asian, Caucasian and South Indian tribes*, Paragraph number 3. This sentence previously stated:

“Although haplogroup U1 is responsible for this contrast, the contribution of M3 in both the Koraga and Jēnu Kurumba (a.k.a. Kāṭṭu Nāyakkar, Kaṭṭunāyakan, cf. **Zvelebil, 1988**) should not be ignored, as this haplogroup is widely present in northwestern populations carrying the Ancestral North Indian component (**Reich et al., 2009**).”

The corrected sentence appears below:

“Although haplogroup U1 is responsible for this contrast, the contribution of M3 in both the Koraga and Jēnu Kuṟumba (a.k.a. Kāṭṭu Nāyakkar, Kaṭṭunāyakan, cf. **Zvelebil, 1988**) should not be ignored, as this haplogroup is widely present in northwestern populations carrying the Ancestral North Indian component (**Reich et al., 2009**).”

A correction has been made to **Discussion**, Paragraph number 4. This sentence previously stated:

“More salient is the exceedingly high frequency of Y-chromosomal haplogroup H1 (M82) in the Koraga (**Anthropological Survey of India, 2021c**), a paternal lineage also found in high frequency in Gōṇḍ tribes (**Sharma, 2009**), in the Kātkarī and Jēnu Kuṟumba and other population groups categorised as untouchable or of low status (**Anthropological Survey of India, 2021c**).”

The corrected sentence appears below:

“More salient is the exceedingly high frequency of Y-chromosomal haplogroup H1 (M82) in the Koraga (**Anthropological Survey of India, 2021c**), a paternal lineage also found in high frequency in Gōṇḍ tribes (**Sharma, 2009**), in the Kātkarī and Jēnu Kuṟumba and other population groups categorised as untouchable or of low status (**Anthropological Survey of India, 2021c**).”

In the published article, there was an error. “Jēnu Kurumba: brief report on a “tribal” language of the Nilgiri area” should be “Jēnu Kuṟumba: Brief report on a “tribal” language of the Nilgiri area.”

A correction has been made to **References**. This sentence previously stated:

“Zvelebil, K. V. (1988). Jēnu Kurumba: brief report on a “tribal” language of the Nilgiri area. J. Am. Orient Soc. 108, 297. doi:10.2307/603656.”

The corrected sentence appears below:

“Zvelebil, K. V. (1988). Jēnu Kuṟumba: Brief report on a “tribal” language of the Nilgiri area. J. Am. Orient Soc. 108, 297. doi:10.2307/603656.”

The authors state that the modifications made do not change the scientific conclusions of the article in any way. The original article has been updated.

