# Supplementary material for: The maternal U1 haplogroup in the Koraga tribe as a correlate of their North Dravidian linguistic affinity
Source: Front Genet. 2024 Feb 7;14:1303628. doi: 10.3389/fgene.2023.1303628 (PMC10880486; doi:10.3389/fgene.2023.1303628)
Supplement: Supplementary file 1 [file DataSheet1.docx]

Supplementary Material

# Supplementary Figures and Tables

## Supplementary Figures

**Supplementary Figure 1.** Intrapopulation Fst matrix showing relationship between the three clans

**
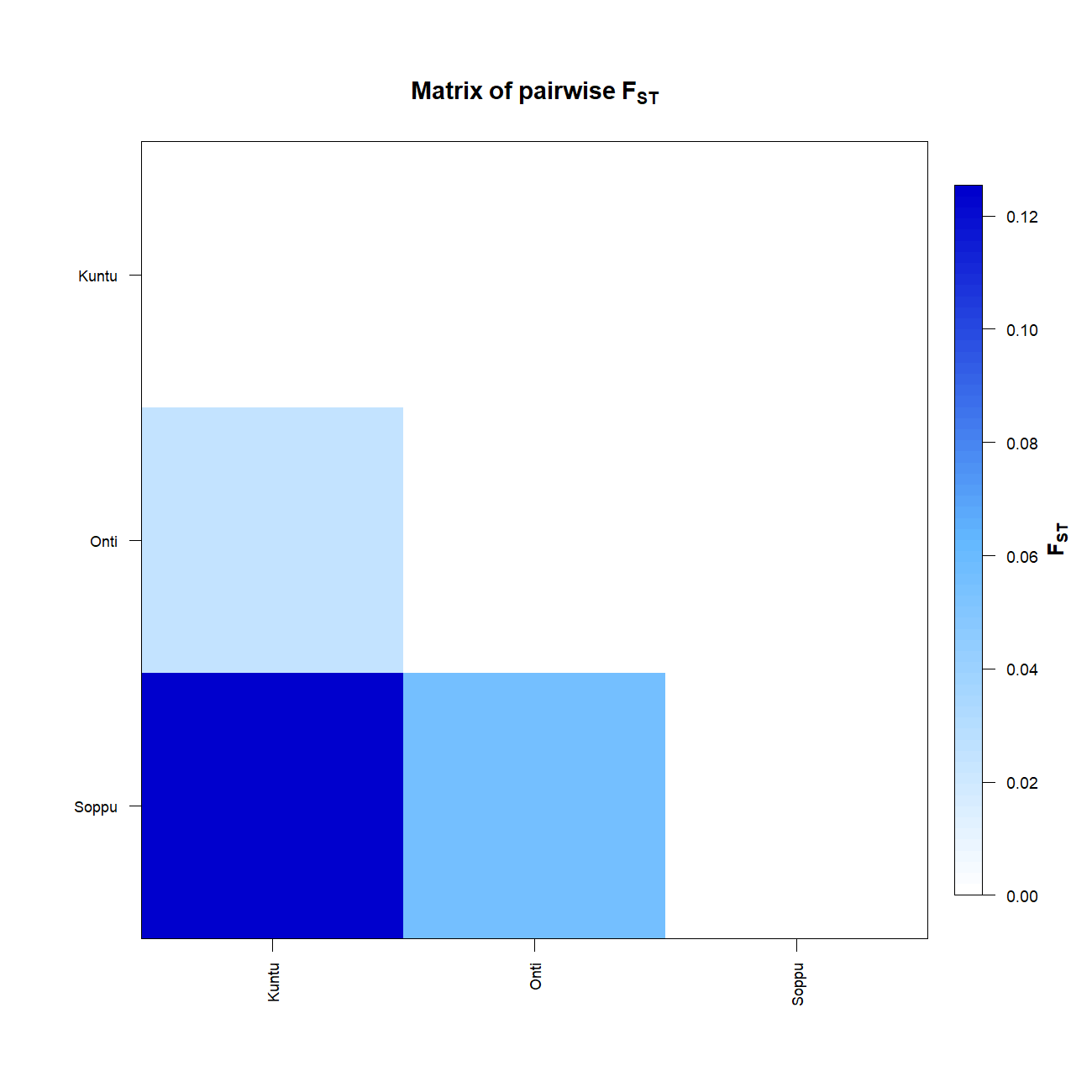
**

Soppu and Kuntu clans are more drifted. Onti and Kuntu are closer than Onti and Soppu.

**Supplementary Figure 2.** Average number of pairwise differences between Koraga and other populations.


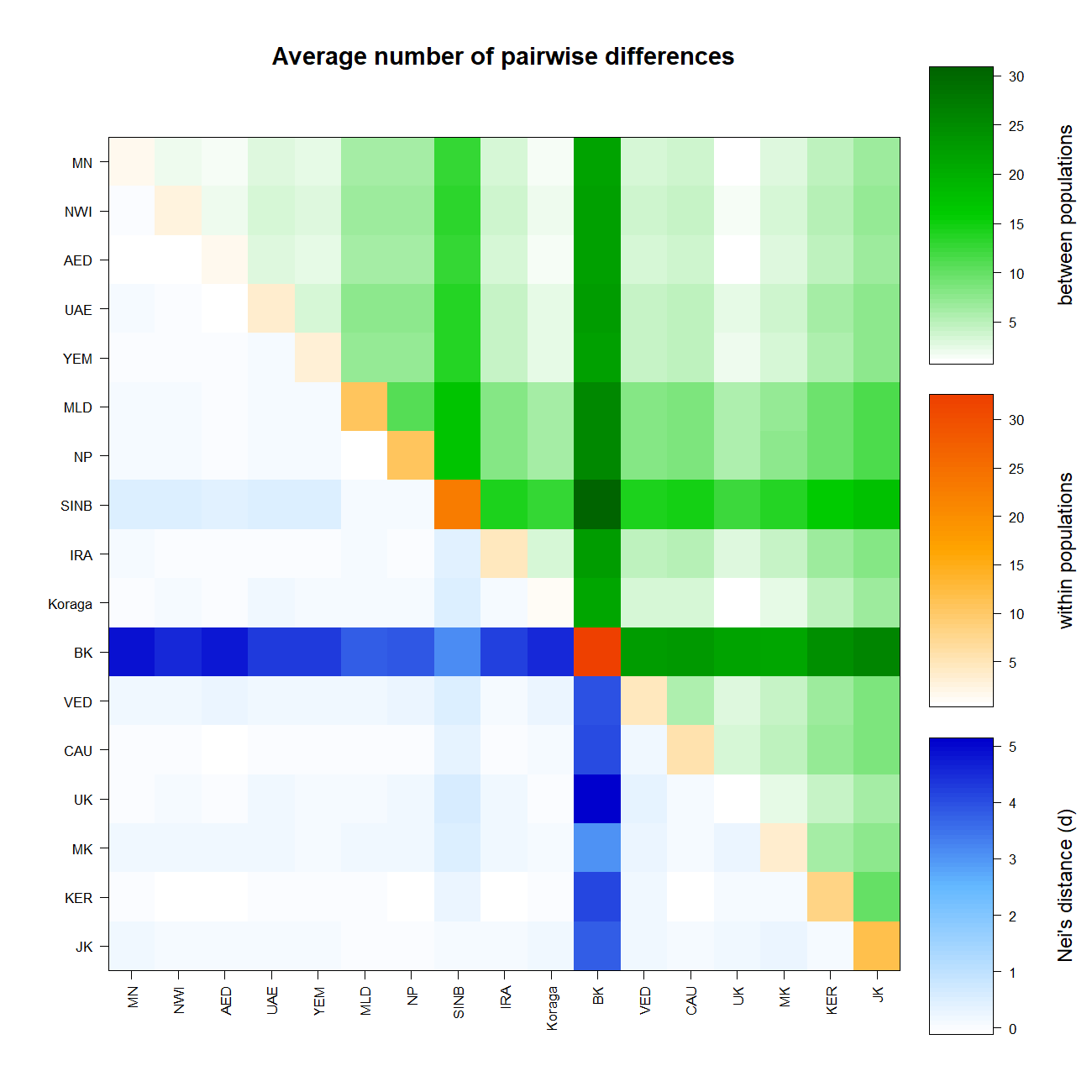


MN: Munda, NWI: North West Indian, AED: Dubai, UAE: United Arab Emirates, YEM: Yemen, MLD: Maldives, NP: North Pakistan, SINB: Sindhi-Balochi, IRA: Iran, BK: Betta Kuruba, VED: Vedda, CAU: Caucasus, UK: Urali Kuruman, MK: Malekudiya, KER: Kerala, JK: Jenu Kuruba

**Supplementary Figure 3.** NJ Tree comparing Koraga, Anatolian, Modern African and Ancient West Eurasian samples.


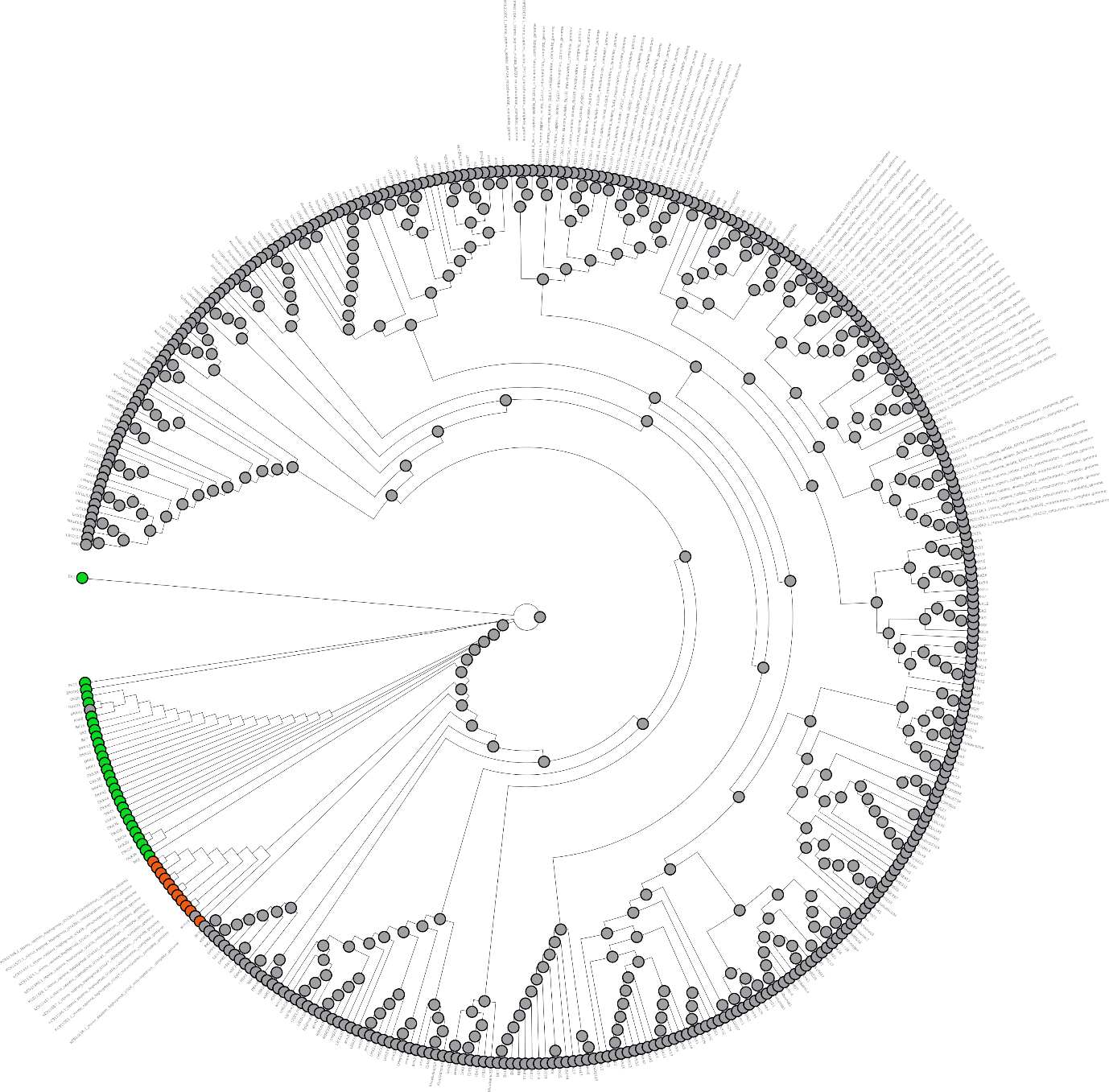


In order to understand the molecular relationship between Koraga and West Eurasian populations we pooled modern and ancient samples from Africa, Anatolia and West Eurasia. Interestingly, the Iranian samples (Orange) cluster along with Koragas (Green) mainly because of U1a haplogroup. This led us to study the divergence time of this haplogroup using Bayesian analysis.

## Supplementary Tables

| **Supplementary Table 1.** Details of mtDNA primers used in the present study | | | | |
| --- | --- | --- | --- | --- |
| **Primer**  **Name** | **Sequence 5'-3'** | **Length** | **3' Position** | **Product length** |
| 22F | TGAAACTTCGGCTCACTCCT | 20 | 14856 |  |
| 22R | AGCTTTGGGTGCTAATGGTG | 20 | 15978 | 1162 |
| 23F | TCATTGGACAAGTAGCATCC | 20 | 15811 |  |
| 23R | GAGTGGTTAATAGGGTGATAG | 21 | 16215 | 765 |
| 24F | CACCATCCTCCGTGAAATCA | 20 | 16420 |  |
| 24R | AGGCTAAGCGTTTTGAGCTG | 20 | 775 | 954 |

**Supplementary Table 2.** Comparison divergence time for U1 haplogroup

| **Clade** | **TMRCA** | **Population/Region** | **Reference** |
| --- | --- | --- | --- |
| U1 | 38,969 | Caucasus and South Asia | **Present Study** |
|  | 38,070 (24,990-51,750) | Iran | (Derenko *et al.*, 2013) |
| U1a | 20,720 (14,740-26,870) | Iran | (Derenko *et al.*, 2013) |
|  | 16,971 (6,919-28,341) | Koraga, South West India | **Present Study** |
| U1a1a4 | 17,900 ± 6800 | South India | (Palanichamy *et al.*, 2015) |
| U1a | 18462.2 | Global | (Behar *et al.*, 2012) |
| U1b | 14,070 (8,590-19,700) | Iran | (Derenko *et al.*, 2013) |
|  | 13664.2 | Global | (Behar *et al.*, 2012) |
|  | 11,955 | Caucasus and Middle East | **Present Study** |

A gradient in the divergence time is observed from west to east clearly suggesting migration of U1 individuals from Iran/Caucasus to South West India. Time to the most recent common ancestor (TMRCA) for South Indian U1 samples falls at the end of the Last glacial maximum ~18 kya.

| **Supplementary Table 3. Mutations observed in the 2031 nucleotide base pair sequence in the D loop covering the HVSI and HVSII region** | | | | | | | | | | | | | | | | | | |
| --- | --- | --- | --- | --- | --- | --- | --- | --- | --- | --- | --- | --- | --- | --- | --- | --- | --- | --- |
| **SampleID** | **Range** | **Haplogroup** | **Polymorphisms** | | | | | | | | | | | | | | | |
| BK1 | 15000-15800;15836-16190;16191-16569;1-500; | U1a | 15148A | 15326G | 15954C | 16189C | 16249C | 16400T | 73G | 199C | 208C | 263G | 285T |  |  |  |  |  |
| BK10 | 15000-15800;15836-16190;16191-16569;1-500; | U1a | 15148A | 15326G | 15954C | 16189C | 16249C | 16400T | 73G | 199C | 263G | 285T |  |  |  |  |  |  |
| BK11 | 15000-15800;15836-16190;16191-16569;1-500; | U1a | 15148A | 15326G | 15954C | 16189C | 16249C | 16400T | 73G | 199C | 208C | 263G | 285T |  |  |  |  |  |
| BK12 | 15000-15800;15836-16190;16191-16569;1-500; | U2a1 | 15326G | 16051G | 16172C | 16206C | 16230G | 16311C | 16519C | 73G | 263G |  |  |  |  |  |  |  |
| BK13 | 15000-15800;15836-16190;16191-16569;1-500; | L3e'i'k'x | 15043A | 15301A | 15326G | 16126C | 16223T | 16519C | 73G | 146C | 150T | 263G |  |  |  |  |  |  |
| BK14 | 15000-15800;15836-16190;16191-16569;1-500; | M30 | 15043A | 15301A | 15326G | 16223T | 16519C | 73G | 195A | 263G | 489C |  |  |  |  |  |  |  |
| BK15 | 15000-15800;15836-16190;16191-16569;1-500; | H33b | 15148A | 15326G | 16249C | 16400T |  |  |  |  |  |  |  |  |  |  |  |  |
| BK16 | 15000-15800;15836-16190;16191-16569;1-500; | U1a | 15148A | 15326G | 15954C | 16189C | 16249C | 16400T | 73G | 199C | 263G | 285T |  |  |  |  |  |  |
| BK17 | 15000-15800;15836-16190;16191-16569;1-500; | U2 | 15326G | 16051G | 16172C | 16400T | 73G | 263G |  |  |  |  |  |  |  |  |  |  |
| BK18 | 15000-15800;15836-16190;16191-16569;1-500; | U2a1 | 15326G | 16051G | 16172C | 16206C | 16230G | 16311C | 16519C | 73G | 263G |  |  |  |  |  |  |  |
| BK19 | 15000-15800;15836-16190;16191-16569;1-500; | U1a | 15148A | 15326G | 15954C | 16189C | 16206C | 16230G | 16311C | 16519C | 73G | 199C | 263G | 285T |  |  |  |  |
| BK2 | 15000-15800;15836-16190;16191-16569;1-500; | U1a | 15148A | 15326G | 15954C | 16189C | 16249C | 16400T | 73G | 199C | 263G | 285T |  |  |  |  |  |  |
| BK3 | 15000-15800;15836-16190;16191-16569;1-500; | M3a2a | 15043A | 15326G | 16126C | 16223T | 16519C | 73G | 146C | 150T | 263G | 482C | 489C |  |  |  |  |  |
| BK4 | 15000-15800;15836-16190;16191-16569;1-500; | U2a1 | 15326G | 16051G | 16172C | 16206C | 16230G | 16311C | 16519C | 73G | 263G |  |  |  |  |  |  |  |
| BK5 | 15000-15800;15836-16190;16191-16569;1-500; | M3a2a | 15043A | 15301A | 15326G | 16126C | 16223T | 16519C | 73G | 146C | 150T | 263G | 482C | 489C |  |  |  |  |
| BK6 | 15000-15800;15836-16190;16191-16569;1-500; | U2a1 | 15326G | 16051G | 16172C | 16206C | 16230G | 16311C | 16519C | 73G | 263G |  |  |  |  |  |  |  |
| BK7 | 15000-15800;15836-16190;16191-16569;1-500; | U1a | 15148A | 15326G | 15954C | 16189C | 16249C | 16400T | 73G | 199C | 263G | 285T |  |  |  |  |  |  |
| BK8 | 15000-15800;15836-16190;16191-16569; | U1a | 15148A | 15326G | 15954C | 16189C | 16249C | 16400T | 73G | 263G |  |  |  |  |  |  |  |  |
| BK9 | 15000-15800;15836-16190;16191-16569;1-500; | U2 | 15326G | 16051G | 16172C | 16230G | 16311C | 16519C | 73G | 263G |  |  |  |  |  |  |  |  |
| DKK1 | 15000-15800;15836-16190;16191-16569;1-500; | M3a2a | 15043A | 15301A | 15326G | 16126C | 16519C | 73G | 146C | 150T | 263G | 482C | 489C |  |  |  |  |  |
| DKK10 | 15000-15800;15836-16190;16191-16569;1-500; | U1a | 15148A | 15326G | 15954C | 16189C | 16400T | 73G | 199C | 208C | 263G | 285T |  |  |  |  |  |  |
| DKK11 | 15000-15800;15836-16190;16191-16569;1-500; | M3a2a | 15043A | 15301A | 15326G | 16126C | 16209C | 16223T | 16519C | 73G | 146C | 150T | 263G | 482C | 489C |  |  |  |
| DKK12 | 15000-15800;15836-16190;16191-16569;1-500; | U1a | 15148A | 15326G | 15954C | 16189C | 16249C | 16400T | 73G | 199C | 263G | 285T |  |  |  |  |  |  |
| DKK13 | 15000-15800;15836-16190;16191-16569;1-500; | U2a1 | 15326G | 16051G | 16172C | 16206C | 16230G | 16311C | 16519C | 73G | 263G |  |  |  |  |  |  |  |
| DKK14 | 15000-15800;15836-16190;16191-16569;1-500; | M7a | 15043A | 15301A | 15326G | 16126C | 16209C | 16223T | 16519C | 73G | 146C | 150T | 263G | 489C |  |  |  |  |
| DKK15 | 15000-15800;15836-16190;16191-16569;1-500; | M3a2a | 15043A | 15301A | 15326G | 16126C | 16223T | 16519C | 73G | 146C | 150T | 263G | 482C | 489C |  |  |  |  |
| DKK16 | 15000-15800;15836-16190;16191-16569;1-500; | U1a | 15148A | 15326G | 15954C | 16189C | 16249C | 16400T | 73G | 199C | 263G | 285T |  |  |  |  |  |  |
| DKK17 | 15000-15800;15836-16190;16191-16569;1-500; | U2a1 | 15326G | 16051G | 16172C | 16206C | 16230G | 16311C | 16519C | 73G | 263G |  |  |  |  |  |  |  |
| DKK18 | 15000-15800;15836-16190;16191-16569;1-500; | U2a1 | 15326G | 16051G | 16172C | 16206C | 16230G | 16311C | 16519C | 73G | 263G |  |  |  |  |  |  |  |
| DKK19 | 15000-15800;15836-16190;16191-16569;1-500; | M3a2a | 15043A | 15301A | 15326G | 16126C | 16209C | 16223T | 16519C | 73G | 146C | 150T | 263G | 482C | 489C |  |  |  |
| DKK2 | 15000-15800;15836-16190;16191-16569;1-500; | U1a | 15148A | 15326G | 15954C | 16189C | 16249C | 16400T | 73G | 199C | 263G | 285T |  |  |  |  |  |  |
| DKK20 | 15000-15800;15836-16190;16191-16569;1-500; | U1a | 15326G | 15954C | 16189C | 16249C | 16400T | 73G | 199C | 263G | 285T |  |  |  |  |  |  |  |
| DKK21 | 15000-15800;15836-16190;16191-16569;1-500; | U2a1 | 15326G | 16051G | 16172C | 16206C | 16230G | 16311C | 16519C | 73G | 263G |  |  |  |  |  |  |  |
| DKK22 | 15000-15800;15836-16190;16191-16569;1-500; | M30 | 15043A | 15301A | 15326G | 15431A | 16223T | 16360T | 16519C | 73G | 195A | 263G | 489C |  |  |  |  |  |
| DKK23 | 15000-15800;15836-16190;16191-16569;1-500; | M3a2a | 15043A | 15301A | 15326G | 16126C | 16223T | 16519C | 73G | 146C | 150T | 263G | 482C | 489C |  |  |  |  |
| DKK24 | 15000-15800;15836-16190;16191-16569;1-500; | U1a | 15148A | 15326G | 15954C | 16189C | 16249C | 16400T | 73G | 263G | 285T |  |  |  |  |  |  |  |
| DKK25 | 15000-15800;15836-16190;16191-16569;1-500; | U2 | 15326G | 16051G | 16172C | 16230G | 16311C | 16519C | 73G | 263G |  |  |  |  |  |  |  |  |
| DKK26 | 15000-15800;15836-16190;16191-16569;1-500; | U1a | 15148A | 15326G | 15954C | 16189C | 16400T | 73G | 199C | 263G | 285T |  |  |  |  |  |  |  |
| DKK27 | 15000-15800;15836-16190;16191-16569;1-500; | M30 | 15043A | 15301A | 15326G | 15431A | 16223T | 16360T | 16519C | 73G | 195A | 263G | 489C |  |  |  |  |  |
| DKK28 | 15000-15800;15836-16190;16191-16569;1-500; | U1a | 15326G | 15954C | 16189C | 16249C | 16400T | 73G | 199C | 263G | 285T |  |  |  |  |  |  |  |
| DKK29 | 15000-15800;15836-16190;16191-16569;1-500; | U1a | 15326G | 15954C | 16189C | 16214T | 16249C | 16400T | 73G | 199C | 204C | 263G | 285T |  |  |  |  |  |
| DKK3 | 15000-15800;15836-16190;16191-16569;1-500; | U1a | 15148A | 15326G | 15954C | 16189C | 16249C | 16400T | 73G | 199C | 263G | 285T |  |  |  |  |  |  |
| DKK30 | 15000-15800;15836-16190;16191-16569;1-500; | N9b | 15326G | 15932C | 15954G | 16145A | 16189C | 16223T | 16463G | 16519C | 73G | 195C | 199C | 263G |  |  |  |  |
| DKK31 | 15000-15800;15836-16190;16191-16569;1-500; | U1a | 15148A | 15326G | 15954C | 16189C | 16222T | 16249C | 16400T | 73G | 199C | 263G | 285T |  |  |  |  |  |
| DKK32 | 15000-15800;15836-16190;16191-16569;1-500; | H2a* | 15326G | 16126C | 16519C | 73G | 146C | 150T | 263G |  |  |  |  |  |  |  |  |  |
| DKK33 | 15000-15800;15836-16190;16191-16569;1-500; | M3a2a | 15043A | 15301A | 15326G | 16126C | 16223T | 16519C | 73G | 146C | 150T | 263G | 482C | 489C |  |  |  |  |
| DKK34 | 15000-15800;15836-16190;16191-16569;1-500; | M30 | 15043A | 15301A | 15326G | 15431A | 16223T | 16360T | 16519C | 73G | 195A | 263G | 489C |  |  |  |  |  |
| DKK35 | 15000-15800;15836-16190;16191-16569;1-500; | U1a | 15148A | 15326G | 15954C | 16189C | 16249C | 16400T | 73G | 199C | 263G | 285T |  |  |  |  |  |  |
| DKK36 | 15000-15800;15836-16190;16191-16569;1-500; | U1a | 15148A | 15326G | 15954C | 16126C | 16189C | 16249C | 16400T | 73G | 199C | 263G | 285T |  |  |  |  |  |
| DKK37 | 15000-15800;15836-16190;16191-16569;1-500; | M30 | 15043A | 15301A | 15326G | 15431A | 16223T | 16360T | 16519C | 73G | 195A | 263G | 489C |  |  |  |  |  |
| DKK38 | 15000-15800;15836-16190;16191-16569;1-500; | U1a | 15148A | 15326G | 15954C | 16189C | 16249C | 16400T | 73G | 199C | 263G | 285T |  |  |  |  |  |  |
| DKK39 | 15000-15800;15836-16190;16191-16569;1-500; | M3a2a | 15043A | 15301A | 15326G | 16126C | 16519C | 73G | 146C | 150T | 263G | 482C | 489C |  |  |  |  |  |
| DKK4 | 15000-15800;15836-16190;16191-16569;1-500; | M3a2a | 15043A | 15301A | 15326G | 16126C | 16209C | 16223T | 16519C | 73G | 146C | 150T | 263G | 482C | 489C |  |  |  |
| DKK40 | 15000-15800;15836-16190;16191-16569;1-500; | M3a2a | 15043A | 15301A | 15326G | 16126C | 16223T | 16519C | 73G | 146C | 150T | 263G | 482C | 489C |  |  |  |  |
| DKK41 | 15000-15800;15836-16190;16191-16569;1-500; | U1a | 15148A | 15326G | 15954C | 16189C | 16222T | 16249C | 16400T | 73G | 199C | 263G | 285T |  |  |  |  |  |
| DKK42 | 15000-15800;15836-16190;16191-16569;1-500; | U1a | 15148A | 15326G | 15954C | 16189C | 16249C | 16400T | 73G | 199C | 263G | 285T |  |  |  |  |  |  |
| DKK43 | 15000-15800;15836-16190;16191-16569;1-500; | U1a | 15148A | 15326G | 15954C | 16189C | 16249C | 16400T | 73G | 199C | 263G | 285T |  |  |  |  |  |  |
| DKK44 | 15000-15800;15836-16190;16191-16569;1-500; | U1a | 15148A | 15326G | 15954C | 16189C | 16249C | 16400T | 73G | 199C | 263G | 285T |  |  |  |  |  |  |
| DKK45 | 15000-15800;15836-16190;16191-16569;1-500; | M3a2a | 15043A | 15301A | 15326G | 16126C | 16519C | 73G | 146C | 150T | 263G | 482C | 489C |  |  |  |  |  |
| DKK46 | 15000-15800;15836-16190;16191-16569;1-500; | M3a2a | 15043A | 15301A | 15326G | 16126C | 16223T | 16519C | 73G | 146C | 150T | 263G | 482C | 489C |  |  |  |  |
| DKK47 | 15000-15800;15836-16190;16191-16569; | M3 | 15043A | 15301A | 15326G | 16126C | 16209C | 16223T | 16519C |  |  |  |  |  |  |  |  |  |
| DKK48 | 15000-15800;15836-16190;16191-16569;1-500; | U1a | 15148A | 15326G | 15954C | 16189C | 16249C | 16400T | 73G | 199C | 263G | 285T |  |  |  |  |  |  |
| DKK49 | 15000-15800;16191-16569; | U2a1 | 15326G | 16206C | 16230G | 16311C | 16519C |  |  |  |  |  |  |  |  |  |  |  |
| DKK5 | 15000-15800;15836-16190;16191-16569;1-500; | U1a | 15148A | 15326G | 15954C | 16189C | 16249C | 16400T | 73G | 199C | 263G | 285T |  |  |  |  |  |  |
| DKK50 | 15000-15800;16191-16569; | M30 | 15043A | 15301A | 15326G | 15431A | 16223T | 16519C |  |  |  |  |  |  |  |  |  |  |
| DKK51 | 15000-15800;16191-16569; | U1 | 15148A | 15326G | 16249C | 16400T | 16519C |  |  |  |  |  |  |  |  |  |  |  |
| DKK52 | 15000-15800;16191-16569; | U1 | 15148A | 15326G | 16249C | 16400T |  |  |  |  |  |  |  |  |  |  |  |  |
| DKK53 | 15000-15800;16191-16569; | U1 | 15148A | 15326G | 16249C | 16400T |  |  |  |  |  |  |  |  |  |  |  |  |
| DKK54 | 15000-15800;16191-16569; | M30 | 15043A | 15301A | 15326G | 15431A | 16223T | 16360T | 16519C |  |  |  |  |  |  |  |  |  |
| DKK55 | 15000-15800;16191-16569; | M30 | 15043A | 15301A | 15326G | 15431A | 16519C |  |  |  |  |  |  |  |  |  |  |  |
| DKK56 | 15000-15800;16191-16569; | M30 | 15043A | 15301A | 15326G | 15431A | 16223T | 16519C |  |  |  |  |  |  |  |  |  |  |
| DKK57 | 15000-15800;16191-16569; | U1 | 15148A | 15326G | 16249C | 16400T |  |  |  |  |  |  |  |  |  |  |  |  |
| DKK6 | 15000-15800;15836-16190;16191-16569;1-500; | M30 | 15043A | 15301A | 15326G | 15431A | 16223T | 16519C | 73G | 195A | 263G | 489C |  |  |  |  |  |  |
| DKK7 | 15000-15800;15836-16190;16191-16569;1-500; | M30 | 15043A | 15301A | 15326G | 15431A | 16223T | 16519C | 73G | 195A | 263G | 489C |  |  |  |  |  |  |
| DKK8 | 15000-15800;15836-16190;16191-16569;1-500; | M40 | 15043A | 15301A | 15326G | 15932C | 15954G | 16145A | 16189C | 16223T | 16463G | 16519C | 73G | 195C | 263G | 489C |  |  |
| DKK9 | 15000-15800;15836-16190;16191-16569;1-500; | M3a2a | 15043A | 15301A | 15326G | 16126C | 16209C | 16223T | 16519C | 73G | 146C | 150T | 263G | 482C | 489C |  |  |  |
| KSK1 | 15000-15800;15836-16569;1-500; | M3a2a | 15043A | 15301A | 15326G | 16126C | 16400T | 73G | 146C | 150T | 263G | 482C | 489C |  |  |  |  |  |
| KSK10 | 15000-15800;15836-16569;1-500; | M2a1a2 | 15043A | 15301A | 15326G | 15670C | 16223T | 16243C | 16270T | 16274A | 16319A | 16352C | 16519C | 73G | 263G | 447G | 489C |  |
| KSK11 | 15000-15800;15836-16569;1-500; | M2a1a2 | 15043A | 15301A | 15326G | 15670C | 16223T | 16243C | 16270T | 16274A | 16319A | 16352C | 16519C | 73G | 263G | 447G | 489C |  |
| KSK12 | 15000-15800;15836-16569;1-300; | M40 | 15043A | 15301A | 15326G | 15721C | 15932C | 15954G | 16145A | 16189C | 16189C | 16223T | 16463G | 16471A | 16519C | 73G |  |  |
| KSK14 | 15000-15800;15836-16569;1-500; | M2a1a2 | 15043A | 15301A | 15326G | 15670C | 16223T | 16243C | 16270T | 16274A | 16319A | 16352C | 16519C | 73G | 263G | 447G | 489C |  |
| KSK15 | 15000-15800;15836-16569;1-500; | M2a1a2 | 15043A | 15301A | 15326G | 15670C | 16223T | 16243C | 16270T | 16274A | 16319A | 16352C | 16519C |  |  |  |  |  |
| KSK16 | 15000-15800;15836-16569;1-300; | U1a | 15148A | 15326G | 15954C | 16189C | 16189C | 16249C | 16400T | 73G | 199C | 263G | 285T |  |  |  |  |  |
| KSK17 | 15000-15800;16189-16569; | M1a3 | 15043A | 15301A | 15326G | 16189C | 16249C | 16400T |  |  |  |  |  |  |  |  |  |  |
| KSK18 | 15000-15800;16189-16569; | U1a | 15148A | 15326G | 16189C | 16249C | 16400T |  |  |  |  |  |  |  |  |  |  |  |
| KSK19 | 15000-15800;16189-16569; | N | 15148A | 15326G | 16223T | 16519C |  |  |  |  |  |  |  |  |  |  |  |  |
| KSK2 | 15000-15800;15836-16569;1-500; | M40 | 15043A | 15301A | 15326G | 15721C | 15932C | 15954G | 16145A | 16189C | 16223T | 16519C | 73G | 195C | 263G | 489C |  |  |
| KSK20 | 15000-15800;16189-16569; | U1a | 15148A | 15326G | 16189C | 16249C | 16400T |  |  |  |  |  |  |  |  |  |  |  |
| KSK21 | 15000-15800;16189-16569; | N | 15148A | 15326G | 16223T | 16519C |  |  |  |  |  |  |  |  |  |  |  |  |
| KSK3 | 15000-15800;15836-16569;1-500; | M3a2a | 15043A | 15301A | 15326G | 16126C | 16189C | 16223T | 16463G | 16519C | 73G | 146C | 150T | 263G | 482C | 489C |  |  |
| KSK4 | 15000-15800;15836-16569;1-500; | U1a | 15148A | 15326G | 15954C | 16189C | 16223T | 16519C | 73G | 199C | 263G | 285T |  |  |  |  |  |  |
| KSK5 | 15000-15800;15836-16569;1-300; | M1a3b1 | 15043A | 15301A | 15326G | 16126C | 16189C | 16249C | 16400T | 73G | 146C |  |  |  |  |  |  |  |
| KSK6 | 15000-15800;15836-16569;1-300; | D4k | 15043A | 15301A | 15326G | 15721C | 16223T | 16519C | 73G | 195C | 263G |  |  |  |  |  |  |  |
| KSK7 | 15000-15800;15836-16569;1-300; | M40 | 15043A | 15301A | 15326G | 15721C | 15932C | 15954G | 16145A | 16189C | 16189C | 16223T | 16463G | 16471A | 16519C | 73G | 195C | 263G |
| KSK8 | 15000-15800;15836-16569;1-300; | U1a | 15148A | 15954C | 16189C | 16189C | 16249C | 16400T | 73G | 199C | 263G | 285T |  |  |  |  |  |  |
| KSK9 | 15000-15800;15836-16569;1-300; | M40 | 15043A | 15301A | 15326G | 15721C | 15932C | 15954G | 16145A | 16189C | 16463G | 16519C | 73G | 195C | 263G | 489C |  |  |

**References:**

Behar, D. M. *et al.* (2012) ‘A “copernican” reassessment of the human mitochondrial DNA tree from its root’, *American Journal of Human Genetics*, 90(4), pp. 675–684. doi: 10.1016/j.ajhg.2012.03.002.

Derenko, M. *et al.* (2013) ‘Complete Mitochondrial DNA Diversity in Iranians’, *PLOS ONE*, 8(11), p. e80673. doi: https://doi.org/10.1371/journal.pone.0080673.

Palanichamy, M. G. *et al.* (2015) ‘West Eurasian mtDNA lineages in India: an insight into the spread of the Dravidian language and the origins of the caste system’, *Human Genetics*, 134(6), pp. 637–647. doi: 10.1007/s00439-015-1547-4.
